# Regular Physical Activity in the Prevention of Post-Transplant Diabetes Mellitus in Patients after Kidney Transplantation

**DOI:** 10.3390/medicina60081210

**Published:** 2024-07-26

**Authors:** Karol Graňák, Matej Vnučák, Monika Beliančinová, Patrícia Kleinová, Tímea Blichová, Margaréta Pytliaková, Ivana Dedinská

**Affiliations:** 1 Transplant-Nephrology Department, University Hospital Martin, Kollárova 2, 036 01 Martin, Slovakia; granak.k@gmail.com (K.G.);; 2 Department of I. Internal Medicine, University Hospital Martin, Jessenius Faculty of Medicine of Comenius University, 03601 Martin, Slovakia; 3 Department of Gastroenterological Internal Medicine, University Hospital Martin, Jessenius Faculty of Medicine of Comenius University, 03601 Martin, Slovakia

**Keywords:** post-transplant diabetes mellitus, physical activity, kidney transplantation

## Abstract

*Background and Objectives*: Post-transplant diabetes mellitus (PTDM) is a significant risk factor for the survival of graft recipients and occurs in 10–30% of patients after kidney transplant (KT). PTDM is associated with premature cardiovascular morbidity and mortality. Weight gain, obesity, and dyslipidemia are strong predictors of PTDM, and by modifying them with an active lifestyle it is possible to reduce the incidence of PTDM and affect the long-term survival of patients and grafts. The aim of our study was to determine the effect of regular physical activity on the development of PTDM and its risk factors in patients after KT. *Materials and Methods*: Participants in the study had to achieve at least 150 min of moderate-intensity physical exertion per week. The study group (*n* = 22) performed aerobic or combined (aerobic + strength) types of sports activities. Monitoring was provided by the sports tracker (Xiaomi Mi Band 4 compatible with the Mi Fit mobile application). The control group consisted of 22 stable patients after KT. Each patient underwent an oral glucose tolerance test (oGTT) at the end of the follow-up. The patients in both groups have the same immunosuppressive protocol. The total duration of the study was 6 months. *Results*: The patients in the study group had significantly more normal oGTT results at 6 months compared to the control group (*p* < 0.0001). In the control group, there were significantly more patients diagnosed with PTDM (*p* = 0.0212) and with pre-diabetic conditions (impaired plasma glucose and impaired glucose tolerance) at 6 months (*p* = 0.0078). *Conclusions*: Regular physical activity after KT provides significant prevention against the development of pre-diabetic conditions and PTDM.

## 1. Introduction

Diabetes mellitus (DM), which occurs after organ transplantation, is a common and serious metabolic complication [1]. Its incidence has increased over the past few decades and remains high. PTDM develops in 10–30% of the cases in the first year after transplantation [2]. The risk depends on the type of transplanted organ, the recipient’s genetic predisposition, and their age. Its occurrence is most often monitored after KT, where its incidence is around 15–30%. Dedinska et al., in screening patients after KT in the Slovak Republic in 2014, identified PTDM in 38.3% of patients according to the valid American Diabetes Association (ADA) criteria [3]. The current definition of PTDM is based on the recommendations of the International Consensus of 2014, and the diagnosis follows the criteria of the ADA and the World Health Organization (WHO) for the diagnosis of type 2 DM and pre-diabetic conditions: fasting blood glucose > 126 mg/dL (7 mmol/L) in more than one case, random glucose > 200 mg/dL (11.1 mmol/L) with symptoms, and glycemia two hours after the administration of 75 g of glucose in the oral glucose tolerance test (oGTT) > 200 mg/dL (11.1 mmol/L) [4,5].

PTDM is a serious risk factor for the survival of recipients and grafts after KT. It is strongly linked to the occurrence of infections and diseases of the cardiovascular system. It is cardiovascular morbidity and mortality that significantly limit the long-term survival of patients after KT [1,2,6,7].

The major risk factors for the development of PTDM are the metabolic side effects of immunosuppressive drugs, post-transplant viral infections, and hypomagnesemia, following traditional risk factors, typical of patients with type 2 DM [8]. In a study of more than 600 patients after primary KT, the authors confirmed that metabolic syndrome (MS) before KT is an independent risk factor for PTDM [9]. In the multicenter study by Dedinska et al., the strongest predictor of PTDM was insulin resistance before KT [3]. Central obesity, another aspect of MS, is associated with high triglycerides, adipocyte-controlled cytokine release, and subclinical inflammation. These all lead to insulin resistance, which increases the risk of developing PTDM [10]. Low adiponectin levels are closely associated with insulin resistance, and significantly increase the risk of developing PTDM regardless of gender, age, and type of immunosuppression [11,12]. In contrast, in obese patients, leptin production increases significantly in the post-transplant period and is significantly associated with the development of PTDM [11]. It is possible to reduce the high incidence of PTDM by influencing only modifiable risk factors, including obesity, associated insulin resistance, and the other components of MS. Low levels of physical activity have even been identified as a major modifiable risk factor for mortality in patients with end-stage chronic kidney disease (CKD) [13]. Many studies have confirmed that regular physical activity has a significant effect on patients with type 2 DM, not only in prevention but also in treatment regimens [14,15]. Therefore, we can expect a similar effect of active lifestyle modification in patients after KT, despite several differences in the pathogenesis of type 2 DM. However, the current literature lacks sufficient data on the importance of physical activity in the prevention of PTDM and, in particular, an objective assessment of its effect.

Recently, in a sample of patients after KT, we confirmed the positive impact of physical activity on the development of insulin resistance and the parameters of glucose and lipid metabolism. Therefore, our aim was to follow up on these favorable results. In this study, we investigated the effect of regular physical activity on the development of PTDM in patients after KT.

## 2. Materials and Methods

We created a pilot prospective intervention-controlled study that included patients after primary deceased-donor or living-donor KT. All the enrolled patients underwent three outpatient check-ups at the Transplant–nephrology department in Martin during the follow-up: at the beginning, at the third, and at the sixth month. The observation period lasted six months.

### 2.1. Inclusion and Exclusion Criteria

The study included collaborating patients who reached the limit at least 3 months after KT. Other inclusion criteria included a good and stable graft function, defined as an estimated glomerular filtration rate (eGFR) of less than 60 mL/min/1.73 m^2^, calculated using the chronic kidney disease—epidemiology collaboration index (CKD-EPI) formula, and stabilized comorbidities. Patients with confirmed DM, PTDM, or known pre-diabetic status (impaired glucose tolerance and elevated fasting glucose); hemoglobin levels < 100 g/L; and age over 65 years were excluded from the study. Each member of the cohort was set up for the same prophylactic immunosuppression (tacrolimus, mycophenolic acid, and prednisone).

### 2.2. Physical Activity and Its Monitoring

After the primary screening according to inclusion criteria (age, graft function, time after KT, hemoglobin level, and maintenance immunosuppression), we randomly chose patients for the intervention group (*n* = 22). We randomly selected participants from a relatively small pool to ensure the equal representation of women and men, considering the size of the transplanted population at our center. Patient selection was not performed by software but by a third party who was not active in the study. Based on the latest ADA recommendations from 2016 for the prevention and treatment of DM and pre-diabetic conditions, the patients in the intervention group were prescribed a limit of at least 150 min of medium-intensity physical activity per week. The second condition was not to have a break for more than 2 days between activities. The patients had to perform an aerobic type of sport (running, brisk walking, cycling, or swimming) or they could combine it with strength training. Each patient received a Xiaomi Mi Band 4 sports bracelet for the detailed monitoring of sports performance parameters, with a compatible Mi Fit mobile application collecting data on activity type, heart rate, energy expenditure, activity duration, and frequency throughout the monitoring period. Power intensity was determined by the percentage of the maximum heart rate (HRmax). Medium-intensity physical activity includes a range of 64 to 76% HRmax, as well as a high-intensity range of 77 to 93% [16]. At each clinic check-up, the investigator revised and sent the data from the Mi Fit mobile application to his or her email. The investigator was available to answer the patients’ study questions by phone or email between checkups. The patients in the control group also met all of the study’s inclusion and exclusion criteria. They were selected to match the patients in the intervention group according to gender and age. They were also checked out at the same intervals during the follow-up, a total of three times. The patients were instructed not to perform any sports activities during the observed period. The activities allowed were to perform basic work at home, around the house, and walk to work or shop. The goal of not reaching the level of physical activity that was prescribed for the intervention group was controlled using The International Physical Activity Questionnaire (IPAQ) at each clinic follow-up. However, neither a continuous monitoring of physical activity nor a prescribed minimum weekly level of training was in place for this patient group.

### 2.3. Recorded Characteristics

For each patient in both groups, we recorded at the baseline of the follow-up: age (years), body weight (kg), waist circumference (cm), body mass index—BMI (kg/m^2^), the underlying cause of renal failure, family history of DM, smoking, history of acute rejection and its type, time since KT (months), type of induction immunosuppression (antithymocyte immunoglobulin or basiliximab), the presence of delayed graft function, significant cytomegalovirus (CMV) replication (cut-off 10,000), and the need for transient discontinuation of mycophenolic acid and treatment with valganciclovir for 21 days. We recorded the following lab values: serum creatinine (umol/L), eGFR according to CKD-EPI (ml/min/1.73 m^2^), vitamin D (ug/L), hemoglobin (g/L), glycemia (mmol/L), glycated hemoglobin—HbA1c (%), immunoreactive insulin (uIU/mL), c-peptide (pmol/L), cholesterol (mmol/L), triacylglycerols (mmol/L), and proteinuria (g/day). Proteinuria was examined from a 24 h urine sample. We used the homeostatic model assessment of insulin resistance (HOMA-IR) index to determine IR. All the patients maintained a stable serum tacrolimus level between 3.0 and 6.0 ng/L during the follow-up period. We recorded the daily dose of prednisone for each control. At 6 months, at the end of the follow-up, each patient underwent oGTT by drinking a solution of 75 g of glucose in 200 mL of water. We measured plasma glucose levels both during fasting and 30 and 120 min after administering the glucose solution. During the test, the examinee sat in calm conditions.

### 2.4. Statistical Analysis

We used MedCalc version 13.1.2, a certified statistical program (MedCalc Software VAT registration number BE 0809 344,640, Member of the International Association of Statistical Computing, Ostend, Belgium). We used parametric (*t*-test) or non-parametric (Mann–Whitney) tests to compare the continuous variables between the groups, and the t2 test and Fisher’s exact test, as appropriate, to analyze associations between the categorical variables. We used logistic regression for multivariate analysis to identify the independent predictors of PTDM. We identified independent risk factors by means of the Cox proportional hazard model. A *p*-value < 0.05 was considered to be statistically significant.

We did not perform sample size calculations because this was a pilot study, and there was no historical data available.

## 3. Results

The study enrolled 44 patients, with 22 patients each in the intervention and control groups. The basic characteristics of the group and laboratory parameters are shown in Table 1 and Table 2. 

**Figure 1:**
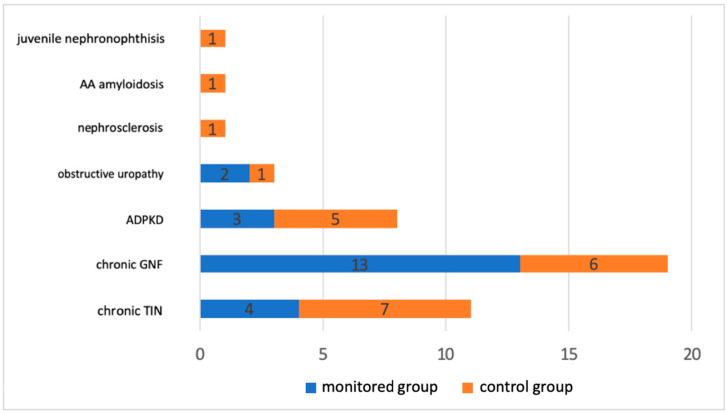
The distribution of the study file according to the cause of renal failure. ADPKD—autosomal dominant polycystic kidney disease; GNF—glomerulonephritis; TIN—tubulointerstitial nephritis.

The age (*p* = 0.9531) and gender (*p* = 0.7677) structures of both groups were not significantly different. The patients forming the control group had an overall shorter time after KT compared to the intervention group (*p* = 0.0002). The mean interval from KT was 15.8 +- 9 months, thus meeting the inclusion criterion (3 months from KT). We did not observe a difference in the daily dose of prednisone or in the use of basiliximab in the induction protocol between the two groups. Vitamin D levels were significantly lower in the control group at the beginning of the follow-up, but this disparity leveled out during the follow-up, and we did not notice it at 6 months. The hemoglobin level was also significantly lower at the beginning, but on average it was in the zone of very mild anemia and thus met the inclusion criterion. In addition, it was saturated during follow-up, and these differences leveled off. Differences in glucose metabolism were also observed. In the intervention group, the fasting plasma glucose levels were significantly lower at baseline (*p* = 0.0045), 3 months (*p* = 0.0016), and 6 months (*p* = 0.0003). However, the higher baseline glycemia in the control group was within the normo-glycemic range and did not represent a pathological condition. We primarily recorded the magnesium levels, but since all the study participants were taking magnesium replacement, their levels were within the physiological range, and there was no difference between the groups. Figure 1 shows the file distribution based on the underlying cause of renal failure. Chronic glomerulonephritis and chronic tubulointerstitial nephritis accounted for the majority.

In an intervention group of 22 patients, 15 practiced isolated aerobic training and 7 combined types of training. Cycling, running, brisk walking, swimming, hiking, and weight training have been practiced.

In addition to comparing the observed parameters between the two groups at the specified time points, we also evaluated the development of these parameters in the individual groups during the study period. We found no significant difference in the development of body weight, BMI, waist circumference, creatinine level, eGFR, proteinuria, vitamin D, hemoglobin, c-peptide, immunoreactive insulin, HbA1c, cholesterol, LDL, HDL, and triacylglycerols for a 6-month follow-up in both groups. On the other hand, we found a significant difference in the fasting glucose (*p* = 0.0227) and HOMA-IR index (*p* = 0.0202) in the control group.

**Figure 2:**
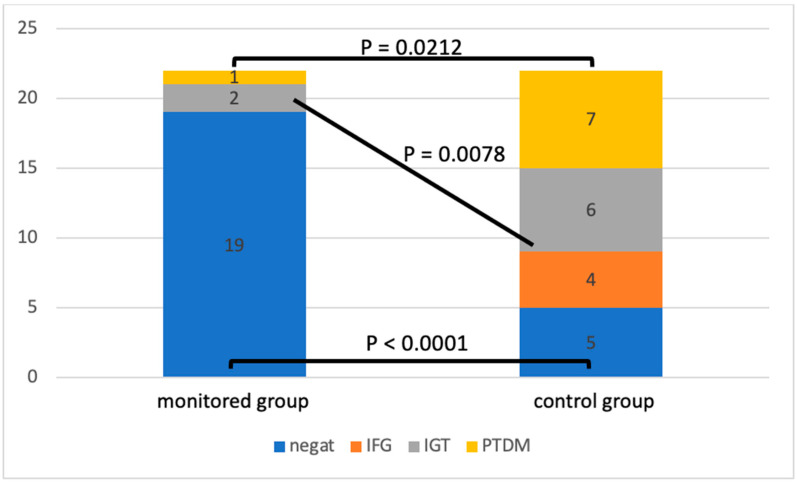
Oral glucose tolerance test results. IFG—impaired fasting glucose, IGT—impaired glucose tolerance, and PTDM—post-transplant diabetes mellitus.

By analyzing the oGTT at the end of the follow-up, we observed differences in the incidence of glucose metabolism disorders (Figure 2). 

**Figure 3:**
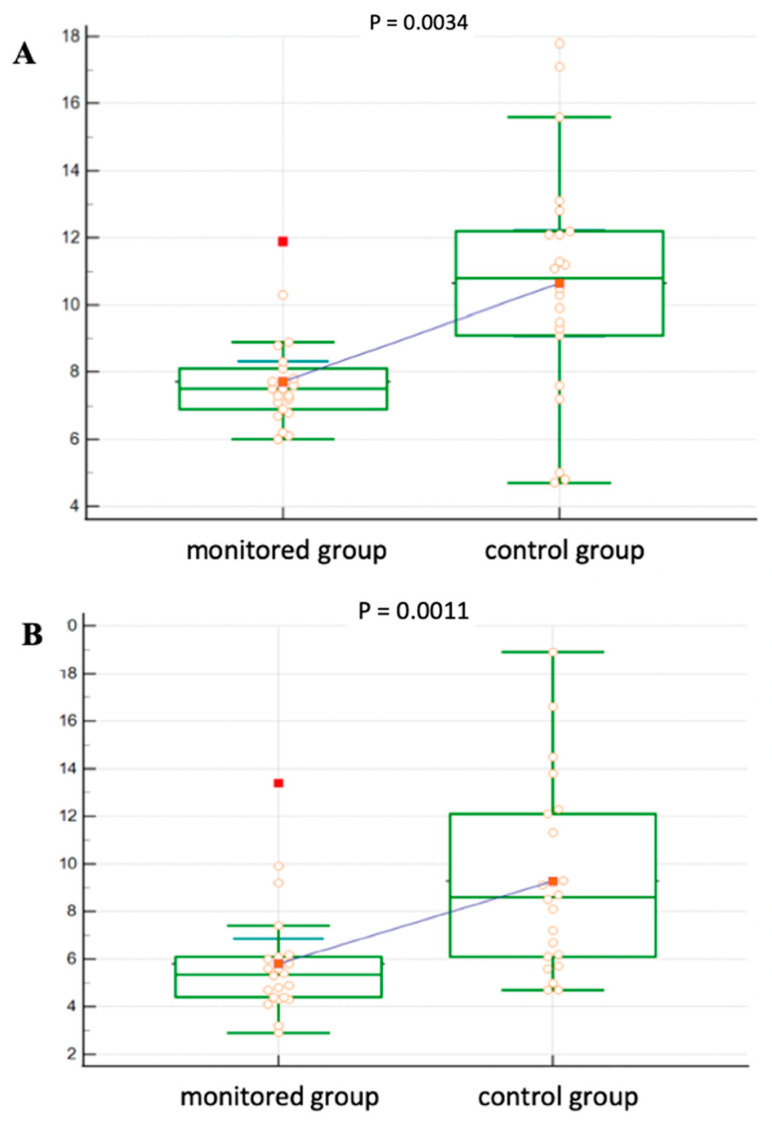
Glycemia during oGTT in both groups, (**A**): 30 min and (**B**): 120 min.

The patients in the control group had significantly fewer physiological oGTT results compared to the intervention group (*p* < 0.0001). In the control group, we also identified a significantly higher incidence of pre-diabetic conditions: impaired glucose tolerance, fasting hyperglycemia (*p* = 0.0078), and diagnosed PTDM (*p* = 0.0212). Figure 3 shows a more detailed analysis of oGTT in both groups. In the control group, the blood glucose level was significantly higher at 30 min of the test (*p* = 0.0034) as well as after 120 min (*p* = 0.0011) compared to the intervention group.

## 4. Discussion

In our study, we discovered that regular physical activity of at least 150 min of moderate intensity per week helped the patients in the intervention group prevent PTDM development. After 6 months of follow-up, the patients in the control group who did not practice such a level of regular physical activity developed significantly more often PTDM, or a pre-diabetic condition (impaired glucose tolerance and fasting hyperglycemia).

Even though there is a lot of clear evidence that exercise can help prevent type 2 diabetes and keep blood sugar levels under control in people who already have it, there is not a lot of information on how important regular physical activity is for patients after KT [16]. In the first intervention study in 2008, Sharif et al. pointed out that active lifestyle modification, including physical training, resulted in improved 2 h postprandial blood glucose levels in patients with already confirmed glucose intolerance [17]. In this case, however, it is more of a therapeutic aspect of using physical activity than a preventive one. In 2015, the authors Karelis et al. conducted a randomized pilot study in patients 6–8 weeks after KT who underwent strength training for 16 weeks. A total of 20 patients, divided 1:1 between the intervention and passive groups, had their physical and cardiometabolic attributes assessed, including the risk of developing PTDM, and completed follow-up. After 16 weeks of follow-up, the group of subjects showed no significant differences in metabolic or anthropometric parameters, and oGTT found no lower incidence of PTDM. This work’s limitation lies in its use of isolated strength training, despite the recommendation for aerobic or combined activity to prevent metabolic disorders. These patients underwent three trainings per week, with only one under the supervision of a gym trainer and the other two undergoing treatment at home using an elastic band, thus lacking an objective of sufficient intensity and duration of training. Additionally, the study included patients who were very shortly after KT, did not have stable serum levels, and were on daily doses of immunosuppressive drugs with frequent adjustments, potentially impacting glucose metabolism parameters [18]. Under the proactive supervision of a team of experts, the randomized prospective study CAVIAR (Comparing Glycemic Benefits of Active Versus Passive Lifestyle Intervention in Kidney Allograft Recipients) demonstrated that post-KT recipients can modify their lifestyle to improve their cardiometabolic risk profile. The study randomly assigned 130 patients to the active and passive intervention groups. A renal dietitian led the active intervention using a behavioral change technique, specifically focusing on adjusting eating habits. Additionally, the dietitian promoted an exercise program to enhance physical activity and encouraged participants to maintain an exercise diary to track their progress. We identified no effect of active intervention on the primary endpoints—insulin secretion, sensitivity, and disposition index—during the 6-month follow-up period. On the other hand, this group showed improvement in secondary endpoints, such as a significant reduction in body weight and fat mass. In this study, the development of PTDM showed a clinically declining trend in the intervened recipients after KT but did not reach statistical significance [19]. In contrast to our study, we did not actively intervene in the dietary regime, instead placing more emphasis on it. Simultaneously, these patients lacked the minimum required level (duration and intensity) of physical training and did not undergo objective monitoring using physical performance-sensing devices. In a prospective monocentric study of a sample of 650 recipients, Byambasukh et al. assessed the effect of physical activity on the development of PTDM, cardiovascular mortality, and overall mortality at least one year after KT. The total follow-up was 5.3 years. Mild (4.0–6.5 MET) to intense (≥6.5 MET) sports activities were applied to the subjects. Mild to intense extracurricular physical activity has been associated with a reduced risk of developing PTDM and cardiovascular and overall mortality, regardless of age, gender, baseline renal parameters, transplant characteristics, and other lifestyle habits. However, after adjusting for immunosuppressive therapy, metabolic and anthropometric parameters, and baseline blood glucose levels, this physical load was no longer associated with PTDM. The authors used a validated SQUASH questionnaire to determine regular physical performance during follow-up. Only leisure sports and activities performed while commuting to work in minutes per week were evaluated. We did not include physical activity that occurred during work. The results also found a relatively high group of inactive patients, up to 38%. The limitation in this case, as in other previous studies, is the patients subjectively assessed physical activity. On the other hand, the strong point is a sufficiently long follow-up [20].

In our study, we followed the 2012 Kidney Disease: Improving Global Outcome (KDIGO) guidelines for CKD investigation and management, which recommend that these patients perform at least 30 min of moderate aerobic physical activity five times per week, adjusting to their cardiovascular health and tolerance [21]. Our findings on how to stop PTDM are in line with what the American Diabetes Association (ADA) says should be done for all high-risk patients and people with prediabetes to stop or delay the development of DM [22]. These PTDM prevention results complement and support the first phase of our study’s evaluation, where we demonstrated a significant effect of regular physical activity on reducing the development of pre-diabetic conditions, insulin resistance, and fasting hyperglycemia. A multivariate analysis using the Cox proportional hazard model demonstrated a significantly higher incidence of insulin resistance, as measured by the HOMA-IR index, in the control group after 6 months of follow-up (*p* = 0.0202), and fasting hyperglycemia after 3 months of follow-up (*p* = 0.0279). Only a univariate comparison revealed the impact on anthropometric parameters (waist circumference) and graft function, as expressed by eGFR, between the two groups [23]. One potential contributing factor to the significant difference in the incidence of PTDM and pre-diabetic conditions could have been the significantly different time since KT. As part of the inclusion criteria, we determined a time of at least 3 months from KT, since by that time all the patients have stable levels, a dose of maintenance immunosuppression, and normal graft function. A recent international consensus of experts in the field of PTDM recommends performing a diagnosis of PTDM 10–13 weeks after KT, which our criteria agree with [24]. On the other hand, a proportion of patients diagnosed early after KT could have a potentially reversible disorder and, in the future, will require confirmation at least 1 year after KT. Upon analyzing the patients, we found that five patients in the control group received a diagnosis within a year, while three others received a diagnosis within 6 months of KT. The second factor could be some degree of predisposition, which we did not identify before the study because of the higher basal glycemia in the control group compared with the intervention group. However, we could not identify potential risks before inclusion because all the patients had physiological fasting glycemia at baseline, and there was no difference in anthropometric parameters or other glucose metabolism parameters (HOMA-IR, immunoreactive insulin, c-peptide, and HbA1c). Furthermore, prior to the study entry, all the enrolled patients had a negative oGTT test, although the study protocol did not include this test. However, the patients did not have this test performed at the same time point, which ranged from 3 to 12 months. The patients who were shortly post-transplant had it performed just before the KT; conversely, some patients who were longer post-transplant may have had an oGTT 12 months ago. Another potential factor may be the unequal level of the physical activity of the patients between the groups before the start of the study, as we did not objectively ascertain their exact average activity before the study. Only at the baseline visit did we ascertain the level of physical activity through an interview, concluding that three-quarters of the participants in both groups did not meet the threshold of 150 min of physical activity per week. The patients in the study group were determined to persevere for the entire period; the investigators kept in regular contact with them and motivated them. Those who did not take up a particular sport (e.g., running, swimming, or cycling) practiced daily brisk walking.

Our study has limitations in terms of the total length of follow-up and the size of the examined sample. On the other hand, given that this is a pilot study evaluating physical activity in a transplanted population by objective measurement using sports bracelets, we consider the number of subjects involved to be appropriate. We need larger, ideal multicenter and randomized studies in the future to confirm these findings. With our findings, we aim to reduce the high incidence of PTDM and other metabolic complications after KT by strictly implementing these measures into the regimen early in the post-transplant period, ideally within 3 months of the operation. Furthermore, we require specific data to formulate lifestyle recommendations for kidney recipients, encompassing not only physical activity but also dietary measures.

## 5. Conclusions

Our study’s findings emphasize the importance of incorporating regular physical activity into preventable measures for postoperative glucose metabolism disorders. Carrying out regular aerobic or combined training lasting at least 150 min a week provided our intervening recipients after KT with significant prevention of PTDM or pre-diabetic conditions after only 6 months.

Post-transplant diabetes mellitus is a serious complication after KT with a negative impact on long-term graft survival as well as cardiovascular and overall mortality. When potential recipients arrive at a KT, they already show signs of metabolic syndrome, which is a significant risk factor for the later onset of PTDM, especially in the field of combination immunosuppressive therapy. It is, therefore, necessary to include this type of non-pharmacological intervention in combination with dietary measures as soon as the patient’s general condition, surgical wound healing, and the development of graft function allow, ideally 3 months after KT.

## Figures and Tables

**Table 1:** Basic group characteristics and anthropometric data.

Group Characteristics	Monitored Group *n* = 22	Control Group *n* = 22	*p*-Value
	Basic group characteristics		
Gender—men (%)	50	54.5	0.7677
Age (years)	42.6 ± 8.8	42.8 ± 13.2	0.9531
Time after KT (M)	60.6 ± 50	15.8 ± 9	**0.0002**
Basiliximab in induction (%)	36.4	31.8	0.7504
Delayed graft function (%)	4.5	4.5	1.0000
DM positive family history (%)	41	45.5	0.7658
Smokers (%)	9	13.6	0.6338
Anamnesis of CMV (%)	13.6	9.1	0.6418
Anamnesis of acute rejection	18	4.5	0.1613
Average prednisone dose (mg/day)	5.9 ± 2.4	5.5 ± 1	0.4745
	Anthropometric data		
Body weight (kg) baseline	75 ± 14.3	77 ± 16.4	0.6686
Body weight (kg) 3 M	74.9 ± 13.8	78.3 ± 16.4	0.4610
Body weight (kg) 6 M	75.1 ± 13.4	79.7 ± 17	0.3246
BMI (kg/m^2^) baseline	25.5 ± 3.2	25.5 ± 3.8	1.0000
BMI (kg/m^2^) 3 M	25.4 ± 3	26 ± 3.7	0.5578
BMI (kg/m^2^) 6 M	25.5 ± 2.9	26.4 ± 4	0.3977
Waist circumference (cm) baseline	90.6 ± 12.4	94.1 ± 12.2	0.3507
Waist circumference (cm) 3 M	89.3 ± 11.5	96.7 ± 12.1	**0.0437**
Waist circumference (cm) 6 M	89.1 ± 11.1	96.7 ± 12.3	**0.0372**
Body height (cm)	171 ± 8.2	173 ± 11.7	0.5150

KT—kidney transplant; DM—diabetes mellitus; CMV—cytomegalovirus; BMI—body mass index; M—month.

**Table 2:** Basic group characteristics—laboratory findings.

	Laboratory Parameters—Graft Function		
Creatinine (µmol/L) baseline	95.1 ± 17.4	101.4 ± 23.3	0.3154
Creatinine (µmol/L) 3 M	98.2 ± 19.2	114.5 ± 30.1	**0.0381**
Creatinine (µmol/L) 6 M	94.5 ± 22.1	110.1 ± 27.1	**0.0425**
eGFR CKD-EPI (ml/min) baseline	76.4 ± 15.5	72.7 ± 18.9	0.4816
eGFR CKD-EPI (ml/min) 3 M	74.1 ± 16	63.4 ± 16.3	**0.0036**
eGFR CKD-EPI (ml/min) 6 M	78.2 ± 17.5	65.7 ± 14.6	**0.0137**
Quantitative proteinuria (g/L) baseline	0.236 ± 0.15	0.220 ± 0.18	0.7503
Quantitative proteinuria (g/L) 3 M	0.220 ± 0.19	0.267 ± 0.29	0.5383
Quantitative proteinuria (g/L) 6 M	0.187 ± 0.13	0.347 ± 0.42	0.0952
Vitamin D (µg/L) baseline	31.9 ± 10.2	23.2 ± 7.2	**0.0022**
Vitamin D (µg/L) 3 M	29.5 ± 8.9	24 ± 8	**0.0369**
Vitamin D (µg/L) 6 M	27.9 ± 8.8	26.5 ± 10	0.6246
Hemoglobin (g/L) baseline	143 ± 12.1	133 ± 15	**0.0193**
Hemoglobin (g/L) 3 M	145 ± 10.3	137 ± 16.5	0.0605
Hemoglobin (g/L) 6 M	146 ± 11.5	142 ± 16.7	0.3601
	Laboratory parameters—glucose metabolism		
Fasting glucose (mmol/L) baseline	4.7 ± 0.6	5.2 ± 0.3	**0.0045**
Fasting glucose (mmol/L) 3 M	4.8 ± 0.6	5.7 ± 1.1	**0.0016**
Fasting glucose (mmol/L) 6 M	4.8 ± 0.6	5.7 ± 0.9	**0.0003**
C—peptide (µg/L) baseline	2.5 ± 1	3.1 ± 1.2	0.0788
C—peptide (µg/L) 3 M	2.2 ± 0.8	2.8 ± 1.1	**0.0447**
C—peptide (µg/L) 6 M	2.4 ± 0.9	4 ± 2	**0.0014**
Immunoreactive insulin—IRI (mU/l) baseline	8.4 ± 6.2	7.8 ± 3.2	0.6887
Immunoreactive insulin—IRI (mU/l) 3 M	8.3 ± 6.8	7.9 ± 3.4	0.8063
Immunoreactive insulin—IRI (mU/l) 6 M	8.7 ± 4.7	9.7 ± 3.7	0.4374
HOMA-IR baseline	1.7 ± 1.3	1.8 ± 0.8	0.7601
HOMA-IR 3 M	1.8 ± 1.5	2.4 ± 1.2	0.1504
HOMA-IR 6 M	1.9 ± 1	2.5 ± 1.1	0.0653
Glycated hemoglobin—HbA1c (%) baseline	3.6 ± 0.4	3.8 ± 0.7	0.2512
Glycated hemoglobin—HbA1c (%) 3 M	3.6 ± 0.5	3.6 ± 0.5	1.0000
Glycated hemoglobin—HbA1c (%) 6 M	3.6 ± 0.5	3.9 ± 0.7	0.1094
	Laboratory parameters—lipid profile		
Cholesterol (mmol/L) baseline	5 ± 0.8	5.3 ± 1	0.2781
Cholesterol (mmol/L) 3 M	4.9 ± 0.8	5.1 ± 1	0.4679
Cholesterol (mmol/L) 6 M	4.8 ± 0.8	5.2 ± 1	0.1504
LDL—low-density lipoprotein (mmol/L) baseline	2.8 ± 0.7	3.2 ± 0.8	0.0848
LDL—low-density lipoprotein (mmol/L) 3 M	2.8 ± 0.7	3 ± 0.9	0.4153
LDL—low-density lipoprotein (mmol/L) 6 M	2.7 ± 0.8	3.2 ± 0.8	**0.0444**
HDL—high-density lipoprotein (mmol/L) baseline	1.5 ± 0.4	1.3 ± 0.4	0.1047
HDL—high-density lipoprotein (mmol/L) 3 M	1.5 ± 0.5	1.4 ± 0.5	0.5107
HDL—high-density lipoprotein (mmol/L) 6 M	1.5 ± 0.5	1.4 ± 0.4	0.4679
TAG—triglycerides (mmol/L) baseline	2 ± 1.4	2.2 ± 1	0.5885
TAG—triglycerides (mmol/L) 3 M	1.5 ± 0.9	1.8 ± 0.7	0.2240
TAG—triglycerides (mmol/L) 6 M	1.7 ± 0.8	2 ± 0.9	0.2492

M—month; eGFR—estimated glomerular filtration rate; CKD-EPI—chronic kidney disease—epidemiology collaboration; HOMA-IR—homeostatic model assessment for insulin resistance, LDL—low-density lipoprotein; HDL—high-density lipoprotein; TAG—triglycerides.

## Data Availability

The data that support the findings of this study are available from the first author upon reasonable request.

## Abbreviations

ADA	American Diabetes Association
BMI	body mass index
CKD	chronic kidney disease
CKD-EPI	chronic kidney disease—epidemiology collaboration index
CMV	cytomegalovirus
DM	diabetes mellitus
eGFR	estimated glomerular filtration rate
FPG	fasting plasma glucose
FPI	fasting plasma insulin
HbA1c	glycated hemoglobin
HOMA-IR	homeostatic model assessment of insulin resistance
HRmax	maximum heart rate
KDIGO	Kidney Disease: Improving Global Outcomes
PTDM	post-transplant diabetes mellitus
KT	kidney transplantation
WHO	World Health Organization
oGTT	oral glucose tolerance test
MS	metabolic syndrome
MET	metabolic equivalent task
